# Long-term outcomes of patients with embolic stroke of undetermined source according to subtype

**DOI:** 10.1038/s41598-024-58292-4

**Published:** 2024-04-23

**Authors:** Il Hyung Lee, JoonNyung Heo, Hyungwoo Lee, JaeWook Jeong, Joonho Kim, Minho Han, Joonsang Yoo, Jinkwon Kim, Minyoul Baik, Hyungjong Park, Jae Wook Jung, Young Dae Kim, Hyo Suk Nam

**Affiliations:** 1 Department of Neurology, National Health Insurance Service Ilsan Hospital, Goyang, Republic of Korea; 2 Department of Neurology, Yonsei University College of Medicine, 50-1 Yonsei-ro, Seodaemun-gu, Seoul, 03722 Republic of Korea; 3 Department of Neurology, Ewha Womans University College of Medicine, Seoul, Republic of Korea; 4 Department of Neurology, Yonsei University College of Medicine, Yongin Severance Hospital, Yongin, Republic of Korea; 5 Department of Neurology, Keimyung University School of Medicine, Daegu, Republic of Korea; 6 Department of Neurology, Kyung Hee University Hospital at Gangdong, Seoul, Republic of Korea

**Keywords:** Neuroscience, Diseases, Medical research, Neurology, Risk factors

## Abstract

The prognosis of patients with embolic stroke of undetermined source (ESUS) may vary according to the underlying cause. Therefore, we aimed to divide ESUS into subtypes and assess the long-term outcomes. Consecutive patients with acute ischemic stroke who underwent a comprehensive workup, including transesophageal echocardiography and prolonged electrocardiography monitoring, were enrolled. We classified ESUS into minor cardioembolic (CE) ESUS, arteriogenic ESUS, two or more causes ESUS, and no cause ESUS. Arteriogenic ESUS was sub-classified into complex aortic plaque (CAP) ESUS and non-stenotic (< 50%) relevant artery plaque (NAP) ESUS. A total of 775 patients were enrolled. During 1286 ± 748 days follow-up, 116 major adverse cardiovascular events (MACE) occurred (4.2 events/100 patient-years). Among the ESUS subtypes, CAP ESUS was associated with the highest MACE frequency (9.7/100 patient-years, p = 0.021). Cox regression analyses showed that CAP ESUS was associated with MACE (hazard ratio 2.466, 95% confidence interval 1.305–4.660) and any stroke recurrence (hazard ratio 2.470, 95% confidence interval, 1.108–5.508). The prognosis of ESUS varies according to the subtype, with CAP ESUS having the worst prognosis. Categorizing ESUS into subtypes could improve patient care and refine clinical trials.

## Introduction

Embolic stroke of undetermined source (ESUS) is defined as a non-lacunar ischemic stroke characterized by the absence of intracranial and extracranial arterial stenoses and major-risk cardioembolic (CE) sources^1,2^. Up to one-quarter of patients with ischemic stroke have ESUS. The associated stroke recurrence rate is approximately 4.5% per year and mortality rate is approximately 5.2% per year^1–5^.

Despite the poor outcomes of patients with ESUS, optimal secondary prevention strategies are not established. Given the undetermined embolic source, anticoagulant therapy is expected to be effective for secondary prevention in these patients. However, in two large randomized controlled trials, direct oral anticoagulants showed no benefit over aspirin in patients with ESUS^6,7^, possibly because of heterogeneity in the embolic source. Patient management can vary according to ESUS subtype. Patients with hidden atrial fibrillation might benefit from anticoagulant therapy, whereas those with < 50% stenosis in the carotid or cerebral arteries and complex aortic plaque (CAP) might benefit from antiplatelet therapy^8,9^. Therefore, predicting the outcome according to the ESUS subtype is important.

To the best of our knowledge, the prognosis according to ESUS subtype has not been fully investigated, especially in patients who have undergone a comprehensive workup including transesophageal echocardiography (TEE)^10^. This study aimed to reveal the long-term outcomes of patients with ESUS according to the subtype.

## Results

**Figure 1:**
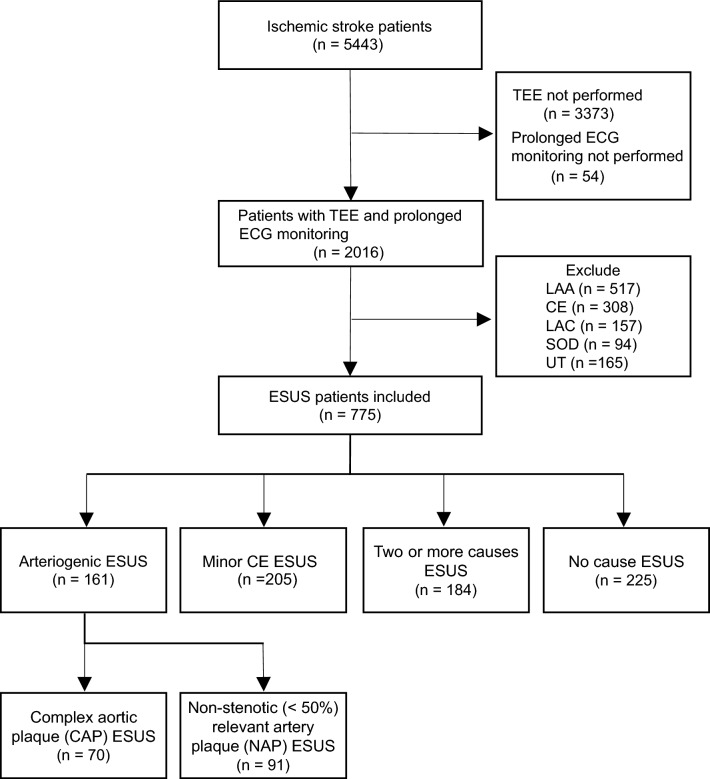
Inclusion and exclusion criteria and ESUS subtypes. Flowchart showing the inclusion and exclusion criteria. Patients who did not undergo transesophageal echocardiography or continuous electrocardiography (ECG) monitoring and those with a stroke other than ESUS were excluded. *ESUS* embolic stroke of undetermined source, *LAA* large-artery atherosclerosis, *CE* cardioembolism, *LAC* lacunar, *SOD* stroke of other determined etiology, *UT* stroke of undetermined etiology with two or more identified causes.

During the study period, 5443 patients with ischemic stroke were registered. Of these, 54 patients who did not undergo continuous ECG monitoring and 3373 patients who did not undergo TEE were excluded. Additionally, 1241 patients with conditions other than ESUS according to the TOAST classification were excluded (517 patients with large-artery atherosclerosis, 308 with cardioembolism, 157 with lacunar infarction, 94 with stroke of other determined etiology, and 165 with stroke of undetermined etiology with two or more causes identified). Finally, 775 (38%) patients were classified as having ESUS, with 161 patients of arteriogenic ESUS (20.8%), 205 patients of minor CE ESUS (26.5%), 184 patients of two or more causes ESUS (23.7%), and 225 patients of no cause ESUS (29.0%). Arteriogenic ESUS was further classified into CAP (70 patients, 9.0%) and NAP (91 patients, 11.7%) ESUS (Fig. 1). Two or more causes ESUS included arteriogenic (CAP and NAP) and minor CE ESUS. The patients with NAP and minor CE causes were 116 (15.0%), CAP and minor CE causes were 43 (5.5%), and CAP, NAP and Minor CE causes were 25 (3.2%) (Supplemental Table 1).

**Table 1:** Demographic characteristics according to the subtype of embolic stroke of undetermined source.

	CAP ESUS (n = 70)	NAP ESUS (n = 91)	Minor CE ESUS (n = 205)	Two or more causes ESUS (n = 184)	No cause ESUS (n = 225)	Total (n = 775)	P value
Age	70.5 ± 10.1	64.8 ± 11.4	59.7 ± 14.1	66.4 ± 12.2	61.4 ± 13.5	63.3 ± 13.3	< 0.001
Sex (men)	53 (75.7)	54 (59.3)	133 (64.9)	117 (63.6)	131 (58.2)	488 (63.0)	0.095
Hypertension	54 (77.1)	78 (85.7)	149 (72.7)	144 (78.3)	134 (59.6)	559 (72.1)	< 0.001
Diabetes	22 (31.4)	35 (38.5)	59 (28.8)	53 (28.8)	48 (21.3)	217 (28.0)	0.034
Hypercholesterolemia	18 (25.7)	21 (23.1)	33 (16.1)	32 (17.4)	40 (17.8)	144 (18.6)	0.322
Current smoker	15 (21.4)	27 (29.7)	60 (29.3)	43 (23.4)	50 (22.2)	195 (25.2)	0.321
Previous stroke	7 (10.0)	16 (17.6)	27 (13.2)	36 (19.6)	27 (12.0)	113 (14.6)	0.134
Congestive heart failure	0 (0.0)	0 (0.0)	8 (3.9)	8 (4.3)	0 (0.0)	16 (2.1)	0.003
Coronary artery disease	23 (32.9)	32 (35.2)	72 (35.1)	88 (47.8)	52 (23.1)	267 (34.5)	< 0.001
Peripheral artery disease	5 (7.1)	5 (5.5)	5 (2.4)	18 (9.8)	6 (2.7)	39 (5.0)	0.005
Initial NIHSS score	2.6 ± 3.6	3.3 ± 3.9	2.8 ± 3.1	3.0 ± 3.2	2.9 ± 4.1	2.9 ± 3.6	0.733
White blood cell, 10^3^/uL	7.75 ± 3.32	7.98 ± 5.06	7.95 ± 2.76	7.91 ± 2.53	7.96 ± 5.19	7.93 ± 3.91	0.996
Platelet, 10^3^/uL	218.5 ± 56.1	240.5 ± 113.6	237.5 ± 67.5	230.3 ± 66.0	241.7 ± 67.0	235.6 ± 73.2	0.143
Total cholesterol, mg/dL	176.0 ± 47.7	191.0 ± 127.7	175.3 ± 44.6	175.0 ± 45.8	183.2 ± 112.1	179.4 ± 82.4	0.488
Triglyceride, mg/dL	143.3 ± 156.3	129.4 ± 93.4	125.3 ± 98.4	123.1 ± 69.9	123.9 ± 80.8	126.5 ± 93.9	0.596
HDL cholesterol, mg/dL	42.3 ± 9.0	43.6 ± 10.5	44.6 ± 11.5	43.6 ± 19.0	43.1 ± 10.2	43.6 ± 13.1	0.684
LDL cholesterol, mg/dL	104.6 ± 38.9	108.7 ± 42.1	104.1 ± 37.5	108.0 ± 37.9	105.6 ± 35.5	106.1 ± 37.7	0.800
Pre-stroke medication							
Antiplatelet	34 (48.6)	28 (30.8)	49 (23.9)	54 (29.3)	55 (24.4)	220 (28.3)	0.001
Anticoagulant	0 (0.0)	0 (0.0)	1 (0.5)	0 (0)	3 (1.3)	4 (0.5)	0.317
Antiplatelet and anticoagulant	0 (0.0)	1 (1.1)	1 (0.5)	2 (1.1)	1 (0.4)	5 (0.6)	0.822
Post-stroke medication							
Antiplatelet	70 (100.0)	91 (100.0)	203 (99.0)	184 (100.0)	225 (100.0)	773 (99.7)	0.233
Anticoagulant	0 (0.0)	0 (0.0)	3 (1.5)	1 (0.5)	5 (2.2)	9 (1.2)	0.301
Statin	68 (97.1)	90 (98.9)	203 (99.0)	182 (98.9)	223 (99.1)	766 (98.8)	0.741

Data are expressed as number (%) or mean ± standard deviation.

*CAP* complex aortic plaque, *ESUS* embolic stroke of undetermined source, *NAP* nonstenotic (< 50%) relevant artery plaque, *NIHSS* National Institutes of Health Stroke Scale, *HDL* High density lipoprotein, *LDL* low density lipoprotein.

### Baseline characteristics

The mean age was 63.3 ± 13.3 years, and 63% were men. The mean NIHSS score was 2.9 ± 3.6. Among all the groups, the patients with CAP ESUS were the oldest (70.0 ± 10.1 years), and hypertension (85.7%) and diabetes (38.5%) were most frequently observed in the patients with NAP ESUS. Patients with two or more causes ESUS had the highest prevalence of congestive heart failure (4.3%), coronary artery occlusive disease (47.8%), and peripheral artery occlusive disease (9.8%) compared to other subtypes. In pre-stroke medications, antiplatelet alone was most frequently used in patients with CAP (p < 0.001). Laboratory test results showed no significant differences between the ESUS subtypes. After index stroke, the secondary prevention was conducted using the antiplatelet (99.7%), anticoagulant (1.2%), and statin (98.8%) (Table 1).

### Prognosis of patients with ESUS

The rates of poor outcomes at 3 months were similar across ESUS subtypes (20.0% for CAP ESUS, 25.3% for NAP ESUS, 16.6% for minor CE ESUS, 14.1% for two or more causes ESUS, and 17.3% for no cause ESUS). Long-term follow-up over 2913 person-years was conducted, with a median of 3.50 years (IQR 2.32–5.25 years). MACEs were observed in 116 patients (4.2/100 patient-years), any stroke recurrence in 69 (2.6/100 patient-years), ischemic stroke recurrence in 54 (2.0/100 patient-years), hemorrhagic stroke in 15 (0.5/100 patient-years), and all-cause mortality in 46 (1.6/100 patient-years). MACE occurred more frequently in patients with CAP ESUS (9.7/100 patient-years) than in those with other ESUS subtypes (NAP ESUS: 4.9/100 patient-years, two or more causes ESUS: 4.3/100 patient-years, no cause ESUS: 4.0/100 patient-years, and minor CE ESUS: 2.8/100 patient-years; p = 0.021; Table 2).

**Table 2:** Short-term and long-term outcomes according to the subtype of embolic stroke of undetermined source.

	CAP ESUS (n = 70)	NAP ESUS (n = 91)	Minor CE ESUS (n = 205)	Two or more causes ESUS (n = 184)	No cause ESUS (n = 225)	Total (n = 775)	P value
Poor outcome at 3 months (mRS 3–6)*	14 (20.0%)	23 (25.3%)	34 (16.6%)	26 (14.1%)	39 (17.3%)	136 (17.5%)	0.225
MACE^†^	9.7	4.9	2.8	4.3	4.0	4.2	0.021
Any stroke recurrence^†^	6.3	2.7	1.8	2.6	2.4	2.6	0.126
Ischemic stroke recurrence^†^	4.6	2.0	1.7	1.6	2.0	2.0	0.380
Hemorrhagic stroke occurrence^†^	1.3	0.6	0.1	0.9	0.4	0.5	0.167
Mortality^†^	3.9	1.5	1.0	1.5	1.6	1.6	0.109

*CAP* complex aortic plaque, *ESUS* embolic stroke of undetermined source, *NAP* nonstenotic (< 50%) relevant artery plaque, *MACE* major adverse cardiovascular event, *mRS* modified Rankin scale, *SD* standard deviation.

*Data are expressed as number (%).

^†^Events are reported as number of events per 100 patient-years.

### Univariable and multivariable analyses of outcomes according to ESUS subtype

**Figure 2:**
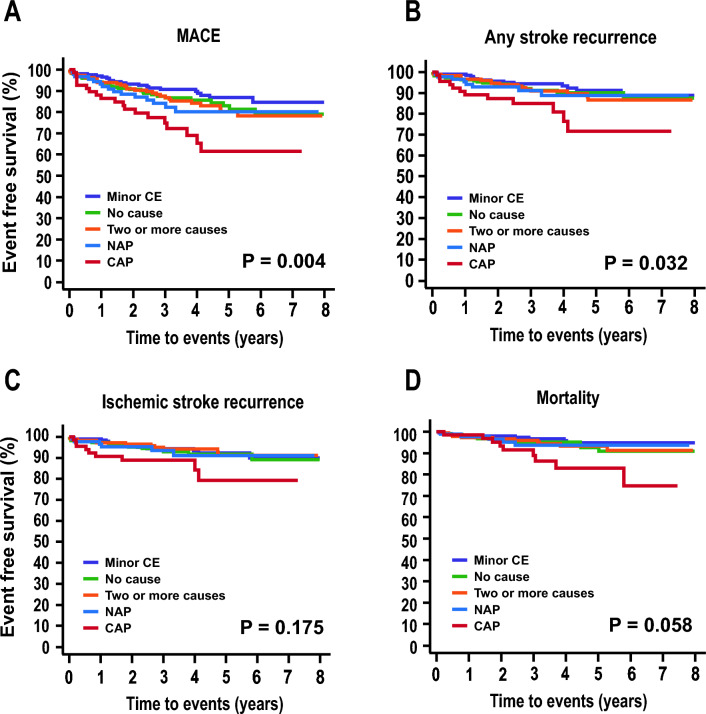
Long-term outcomes during follow-up. Kaplan–Meier survival curves of MACE (**A**), any stroke recurrence (**B**), ischemic stroke recurrence (**C**), and all-cause mortality (**D**). Patients with complex aortic plaques show the highest rates of MACE and any stroke recurrence. However, ischemic stroke recurrence (p = 0.175) and all-cause mortality (p = 0.058) rates were similar across subtypes. *MACE* major adverse cardiovascular event, *CE* cardioembolism, *NAP* non-stenotic (< 50%) relevant artery plaque.

Kaplan–Meier survival analysis revealed that MACE (p = 0.004) and any stroke recurrence rates (p = 0.032) differed according to ESUS subtype. Patients with CAP ESUS showed the highest MACE and any stroke recurrence rates. However, the ischemic stroke recurrence (p = 0.175) and all-cause mortality (p = 0.058) rates were similar across subtypes (Fig. 2).

Univariable analysis revealed that CAP ESUS was associated with MACE along with age, sex, hypertension, diabetes, previous stroke history and platelet count. Multivariable Cox regression analysis revealed that CAP ESUS was independently associated with MACE (HR 2.466, 95% CI 1.305–4.660; Table 3) and any stroke recurrence (HR 2.470, 95% CI 1.108–5.508; Table 4). However, no significant associations were found between CAP ESUS and ischemic stroke recurrence, hemorrhagic stroke, and all-cause mortality (Supplemental Tables 2–4).s

**Table 3:** Univariate and multivariate analyses of the factor associated with major adverse cardiovascular events.

	Univariable analysis		Multivariable analysis	
	HR (95% CI)	P value	HR (95% CI)	P value
Demographics				
Age, year	1.030 (1.014–1.046)	< 0.001	1.019 (1.001–1.036	0.033
Sex, men	1.533 (1.024–2.294)	0.038	1.289 (0.844–1.969)	0.240
Risk factors				
Hypertension	1.665 (1.062–2.610)	0.026	1.225 (0.760–1.976)	0.405
Diabetes	1.735 (1.192–2.525)	0.004	1.486 (1.007–2.194)	0.046
Hypercholesterolemia	1.305 (0.838–2.033)	0.240		
Current smoker	0.720 (0.456–1.137)	0.159		
Previous stroke	2.322 (1.539–3.504)	< 0.001	1.904 (1.243–2.917)	0.003
Initial NIHSS score	1.041(0.996–1.088)	0.073		
Laboratory findings				
White blood cell	1.000 (1.000–1.000)	0.835		
Platelet	0.996 (0.993–0.999)	0.006	0.997 (0.994–1.000)	0.086
Total cholesterol	0.999(0.995–1.002)	0.487		
Triglyceride	1.000 (0.998–1.002)	0.771		
HDL	0.988 (0.971–1.006)	0.181		
LDL	0.998 (0.993–1.003)	0.474		
ESUS classification				
Minor cardioembolic source	Ref	0.006	Ref	0.069
No cause	1.413 (0.818–2.441)	0.215	1.470 (0.845–2.557)	0.173
Two or more causes	1.502 (0.863–2.615)	0.150	1.216 (0.691–2.139)	0.498
Non-stenotic (< 50%) relevant artery plaque	1.700 (0.882–3.278)	0.113	1.326 (0.683–2.574)	0.405
Complex aortic plaque	3.205 (1.732–5.931)	< 0.001	2.466 (1.305–4.660)	0.005

Cox proportional hazards regression analysis was conducted after adjusting for variables that were significant (p < 0.05) in the univariable analysis along with the ESUS subtype.

*HR* hazard ratio, *CI* confidence interval, *NIHSS* National Institutes of Health Stroke Scale, *HDL* high-density lipoprotein cholesterol, *LDL* low-density lipoprotein cholesterol, *Ref* reference.

**Table 4:** Univariate and multivariate analyses of the factor associated with any stroke recurrence.

	Univariable analysis		Multivariable analysis	
	HR (95% CI)	P value	HR (95% CI)	P value
Demographics				
Age, year	1.029 (1.009–1.050)	0.005	1.016 (0.994–1.039	0.153
Sex, men	1.814 (1.049–3.137)	0.033	1.386 (0.780–2.463)	0.265
Risk factors				
Hypertension	1.441 (0.823–2.523)	0.200		
Diabetes	1.806 (1.113–2.929)	0.017	1.505 (0.914–2.478)	0.108
Hypercholesterolemia	1.195 (0.664–2.150)	0.553		
Current smoker	0.709 (0.394–1.276)	0.252		
Previous stroke	3.480 (2.119–5.714)	< 0.001	2.884 (1.716–4.847)	< 0.001
Initial NIHSS score	0.984(0.912–1.062)	0.679		
Laboratory findings				
White blood cell	1.000 (1.000–1.000)	0.592		
Platelet	0.994 (0.990–0.998)	0.007	0.996 (0.992–1.001)	0.093
Total cholesterol	0.998(0.993–1.003)	0.504		
Triglyceride	1.001 (0.999–1.003)	0.464		
HDL	0.979 (0.956–1.003)	0.083		
LDL	0.997 (0.990–1.004)	0.386		
ESUS classification				
Minor cardioembolic source	Ref	0.044	Ref	0.177
No cause	1.281 (0.637–2.577)	0.487	1.292 (0.639–2.613)	0.475
Two or more causes	1.389 (0.685–2.819)	0.362	1.084 (0.528–2.229)	0.825
Non-stenotic (< 50%) relevant artery plaque	1.433 (0.601–3.417)	0.417	1.056 (0.439–2.543)	0.903
Complex aortic plaque	3.220 (1.486–6.977)	0.003	2.470 (1.108–5.508)	0.027

Cox proportional hazards regression analysis was conducted after adjusting for variables that were significant (p < 0.05) in the univariable analysis along with the ESUS subtype.

*HR* hazard ratio, *C*I confidence interval, *NIHSS* National Institutes of Health Stroke Scale, *HDL* high-density lipoprotein cholesterol, *LDL* low-density lipoprotein cholesterol, *Ref* reference.

#### Predictive value of the ESUS subtype classification for classical risk factors

When comparing the predictive value of the ESUS subtype classification and classical risk factors, the p-value of integrated discrimination index (IDI) of median follow up was 0.016 which was statistically significant. Additionally, the overall p-values are less than 0.2, indicating a tendency for differences between the two models (Supplemental Table 5).

## Discussion

In this study, we categorized ESUS into subtypes and found that the prognosis differed among the subtypes. The worst long-term outcomes were observed in patients with CAP ESUS, whereas the best outcomes were observed in patients with minor CE ESUS. After adjusting for covariates, CAP ESUS was independently associated with MACE and any stroke recurrence.

We found that the prognosis of ESUS differed according to the ESUS subtype. Although no differences were observed in the short-term functional outcome at 3 months, the long-term outcomes were significantly different among the ESUS subtypes. MACE occurred most frequently in patients with CAP ESUS, whereas favorable long-term outcomes were most noted in patients with minor CE ESUS. Patients with CAP ESUS experienced three times more MACE during the follow-up period than those with minor CE ESUS.

Secondary prevention of ESUS is not well established. Direct oral anticoagulants have been failed to show significant benefits^6,7^. These results may be due to heterogeneity in ESUS subgroups. Studies including all ESUS subtypes may have attenuated the effects of the study drugs^8,9^. Treatments targeting hidden paroxysmal atrial fibrillation might differ from those targeting other embolic sources, including minor CE sources, < 50% stenosis in the cerebral arteries, and CAP. Some patients with a patent foramen ovale may benefit from closure. If the primary embolic sources are white thrombi forming on the aortic plaque or cerebral arteries, prevention with antiplatelet and statin might be beneficial^11^. To date, no detailed research has been conducted on the prognosis according to ESUS subtype. For patient management and clinical trial design, it is crucial to identify the ESUS subtype and determine the prognosis accordingly.

An exploratory analysis revealed that 8% of the participants of the NAVIGATE ESUS trial had CAP ESUS, with an ischemic stroke recurrence rate of 7.2/100 patient-years^12^. The ischemic stroke recurrence rate in our study (4.6/100 patient-years) was lower than that in the NAVIGATE ESUS trial, possibly due to different treatment strategy. In our study, all patients with CAP ESUS received antiplatelet therapy, and 97.1% received statin. The Aortic Arch Related Cerebral Hazard (ARCH) Trial investigated the superiority of aspirin plus clopidogrel over warfarin. The ARCH trial failed the difference between treatments. Recurrent stroke or vascular events occurred in 7.6% of patients on aspirin plus clopidogrel and 11.3% of patients on warfarin during a median follow-up 3.4 years^13^. Until now, the advantages of these two treatments are still unclear. However, current guidelines recommend antiplatelet therapy and intensive lipid-lowering to prevent recurrent stroke in patients with aortic arch atheroma^14^. Because our study was not a clinical trial and did not intend to test the effects of drugs, we cannot provide the optimal management of aortic atheroma.

Our findings and those of previous studies suggest several hypotheses for the poor prognosis of patients with CAP ESUS. First, the prevalence of CAP ESUS increases with age^12^, and patients with CAP have many risk factors^15^, which might influence the long-term outcomes. Second, CAP has been found to be associated with intracranial atherosclerosis and small vessel disease^15,16^, suggesting that it is a marker of systemic atherosclerosis^15^. Polyvascular disease is associated with poor prognosis in patients with ischemic stroke. Third, CAP may be accompanied by atrial fibrillation^17,18^, and the burden of additional embolic sources may result in a poor prognosis^17^. Therefore, evaluation by TEE to detect CAP in ESUS patients might be helpful in tailoring treatment of ESUS.

This study has several limitations. First, although we included consecutive patients with ESUS, we excluded those who did not undergo a comprehensive workup including TEE and continuous ECG monitoring. ESUS working group investigators recommended a comprehensive stroke workup, but TEE and continuous ECG monitoring were not included as mandatory investigations^4^. In more than half of cases, TEE examination can uncover embolic sources from heart or aorta that can significantly impact the prognosis of patients with ESUS^19^. Second, the effects of clinical characteristics of the ESUS subtypes, such as infarction patterns, vascular territory, and clinical symptoms, were not investigated^20,21^. Since the main purpose of this study was to compare the prognosis according to ESUS subtype, further investigations are needed to evaluate the clinical characteristics of each ESUS subtype. Third, the response to antithrombotic therapy for each ESUS subtype could not be determined in our study because most patients were treated with antiplatelet therapy and lipid-lowering agents.

## Conclusions

We found that prognosis differed according to ESUS subtype. The long-term outcomes of MACE and any stroke recurrence were the worst in patients with CAP ESUS and the best in those with minor CE ESUS. Sub-classifying ESUS and predicting outcomes based on subtypes may improve patient management and clinical trial design.

## Methods

### Study population

Consecutive patients with acute ischemic stroke who were prospectively registered in the Yonsei Stroke Registry^22^ between January 2012 and December 2018 were enrolled. All patients underwent brain magnetic resonance imaging (MRI) and/or computed tomography (CT). Cerebral vessels were evaluated using cerebral angiography (MRI, CT, or digital subtraction angiography). Systemic evaluations included chest radiography, 12-lead electrocardiography (ECG), routine blood tests, and lipid profiling. Cardiac CT were performed in selected patients. To accurately classify ESUS subtypes, we only enrolled patients who had undergone continuous ECG monitoring and TEE. Most patients were admitted to the stroke unit and were continuously monitored with ECG during their stay. Continuous ECG monitoring was also obtained by Holter monitoring and an implantable loop recorder. TEE was part of the standard evaluation but was not performed in patients with a poor general condition, mental decline, impending brain herniation, or an unacceptable esophageal transducer due to swallowing difficulties or tracheal intubation, and in those who did not provide informed consent (Supplemental Table 6)^23^.

ESUS was defined based on the criteria proposed by the Cryptogenic Stroke/ESUS International Working Group^4^. A diagnosis of ESUS was made if the patient exhibited non-lacunar stroke, had no more than 50% stenosis of the relevant proximal artery, and presented with no significant CE source. These sources include atrial fibrillation, atrial flutter, sick sinus syndrome, mechanical or bioprosthetic heart valves, mitral stenosis (with or without atrial fibrillation), left atrial or atrial appendage thrombosis, left ventricular thrombus, akinetic left ventricular segment, recent myocardial infarction (occurring less than 4 weeks ago), dilated cardiomyopathy, atrial myxoma, infective endocarditis, and nonbacterial thrombotic endocarditis. Furthermore, patients with ESUS should not have other rare stroke causes such as reversible cerebral artery vasoconstriction syndrome, antiphospholipid syndrome, dissection, moyamoya disease, or cancer related stroke.

According to its potential cause, we classified ESUS into arteriogenic ESUS, minor CE ESUS, two or more causes ESUS, and no cause ESUS. Arteriogenic ESUS was sub-classified into CAP ESUS and non-stenotic (< 50%) relevant artery plaque (NAP) ESUS^24,25^. Minor CE sources included patent foramen ovale, atrial septal defect, atrial septal aneurysm, congestive heart failure, mitral valve prolapse, mitral annular calcification, left atrial turbulence (smoke), hypokinesia of the left ventricular segment, and myocardial infarction (> 4 weeks but < 6 months)^4,26–28^. CAP was defined by the presence of a plaque in the ascending aorta or aortic arch, which is either 4 mm or more in thickness or contains ulcerated or mobile components (Supplemental Table 1)^29,30^. The Trial of ORG 10172 in Acute Stroke Treatment (TOAST) classification was determined during weekly conferences through the consensus of three stroke neurologists^28,31^.

### Clinical variables

We recorded the following clinical data of each participant: demographic characteristics (age and sex), risk factors (hypertension, diabetes, hypercholesterolemia, current smoking, coronary artery occlusive disease, peripheral artery occlusive disease, and previous stroke status), the initial National Institutes of Health Stroke Scale (NIHSS) score, and laboratory findings (white blood cell count, platelet count, total cholesterol level, triglyceride level, high-density lipoprotein cholesterol level, and low-density lipoprotein cholesterol level). Detailed information is available in a previous publication^31^.

### Follow-up and outcomes

Neurologists and clinical research assistants collected the follow-up information at the outpatient clinic and/or by telephone interview using a structured questionnaire at 3 months, 1 year, and annually thereafter. The mRS score was determined by a structured interview using the Korean version of the mRS (http://stroke-edu.or.kr). A poor outcome at the 3-month follow-up was defined as a modified Rankin scale (mRS) score ≥ 3. Stroke recurrence was defined as a new stroke event occurring > 7 days after the index stroke. Major adverse cardiovascular event (MACE) included ischemic stroke recurrence, hemorrhagic stroke, acute coronary syndrome, heart failure, and all-cause mortality. The censoring date was December 31, 2019. If a patient’s last visit occurred before this date, the date of the last visit was considered the censoring date.

The study was approved by the relevant institutional review board of Severance Hospital of Yonsei University Health System, and the requirement for informed consent was waived because of the retrospective nature of the study (4-2021-1724). The original prospective hospital-based observational study was approved by the institutional review board (4-2007-0389) and informed consent was obtained from the patients or legal representatives. The study was performed in accordance with the World Medical Association Declaration of Helsinki.

### Statistical analyses

Differences between ESUS subtypes were assessed using the chi-square test for categorical variables and independent two-sample t-test or Mann–Whitney U-test for continuous variables. Continuous variables are presented as either the mean with standard deviation or median with interquartile range (IQR). Categorical variables are presented as counts with percentages (%). Kaplan–Meier curves were constructed and the differences across ESUS subtypes were analyzed using the log-rank test. The results were evaluated by considering both statistical and clinical aspects using effect size (mean, proportion, HR), 95% CI and p-value. We assessed how the addition of the ESUS subtype variable to classical risk factors impacts the predictive accuracy of the Cox hazard model for outcomes. We compared the model of classical risk factors alone with model of novel. We calculated Harrell's c-index, Heagerty's area under the curve (AUC), the net reclassification index (NRI), and the integrated discrimination improvement (IDI). Statistical analyses were performed using SPSS for Windows, version 26 (IBM Corp., Armonk, NY, USA), R version 4.3.2 (R Foundation for Statistical Computing, Vienna, Austria) and MedCalc Statistical Software, version 20.026 (MedCalc Software Ltd., Ostend, Belgium; https://www.medcalc.org).

### Supplementary Information

Supplementary Tables.

## Data Availability

The datasets used and/or analyzed during the current study available from the corresponding author on reasonable request.
