# Supplementary material for: Long-term outcomes of patients with embolic stroke of undetermined source according to subtype
Source: Sci Rep. 2024 Apr 23;14:9295. doi: 10.1038/s41598-024-58292-4 (PMC11039691; doi:10.1038/s41598-024-58292-4)
Supplement: Supplementary file 1 — Supplementary Tables. [file 41598_2024_58292_MOESM1_ESM.docx]

**Supplemental Table 1. Details of ESUS subgroup**

| **Details of ESUS** | Number (%) |
| --- | --- |
| CAP ESUS (n = 70) |  |
| Only plaque thickness ≥4 mm | 65 (8.4%) |
| Both plaque thickness ≥4 mm and mobile | 1 (0.1%) |
| Both plaque thickness ≥4 mm and ulcerated | 3 (0.4%) |
| Both plaque thickness ≥4 mm and mobile, and ulcerated | 1 (0.1%) |
|  |  |
| Minor CE ESUS (n = 205) |  |
| PFO | 156 (20.1%) |
| Hypokinesia of the left ventricular segmen | 8 (1.0%) |
| Left atrial turbulence | 4 (0.5%) |
| Atrial septal defect | 4 (0.5%) |
| Atrial septal aneurysm | 3 (0.4%) |
| Mitral annular calcification | 3 (0.4%) |
| Congestive heart failure | 2 (0.3%) |
| Mitral valve prolapse | 0 |
| Myocardial infarction (>4 weeks but <6 months) | 0 |
| Multiple minor CE ESUS | 25 (3.2%) |
|  |  |
| Two or more causes ESUS (n = 184) |  |
| NAP + minor CE | 116 (15.0%) |
| CAP + minor CE | 43 (5.5%) |
| NAP + CAP + minor CE | 25 (3.2%) |

ESUS = Embolic stroke of undetermined source

CAP = Complex aortic plaque

NAP = Nonstenotic atherosclerotic plaque

PFO = Patent foramen ovale

# Supplemental Table 2. Univariable and multivariable analysis for the factor associated with ischemic stroke recurrence

|  | Univariable analysis | | Multivariable analysis | |
| --- | --- | --- | --- | --- |
|  | HR (95% CI) | P value | HR (95% CI) | P value |
| Demographics |  |  |  |  |
| Age, year | 1.033 (1.010–1.057) | 0.005 | 1.025 (1.000–1.052) | 0.052 |
| Sex, men | 2.314 (1.193–4.487) | 0.013 | 1.897 (0.949–3.792) | 0.070 |
| Risk factors |  |  |  |  |
| Hypertension | 1.517 (0.798–2.884) | 0.204 |  |  |
| Diabetes | 1.784 (1.032–3.084) | 0.038 | 1.454 (0.828–2.551) | 0.192 |
| Dyslipidemia | 1.190 (0.613–2.310) | 0.607 |  |  |
| Current smoker | 0.891 (0.477–1.663) | 0.717 |  |  |
| Previous stroke | 3.216 (1.825–5.666) | < 0.001 | 2.617 (1.449–4.725) | 0.001 |
| Initial NIHSS score | 0.941 (0.849–1.042) | 0.241 |  |  |
| Laboratory findings |  |  |  |  |
| White blood cell | 1.000 (1.000–1.000) | 0.953 |  |  |
| Platelet | 0.995 (0.990–0.999) | 0.021 | 0.997 (0.993–1.002) | 0.247 |
| Total cholesterol | 0.999 (0.994–1.004) | 0.641 |  |  |
| Triglyceride | 1.001 (0.999–1.004) | 0.338 |  |  |
| HDL | 0.975 (0.949–1.002) | 0.067 |  |  |
| LDL | 0.998 (0.991–1.006) | 0.653 |  |  |
| ESUS classification |  |  |  |  |
| Minor cardioembolic source | Ref. | 0.201 | Ref. | 0.404 |
| No cause | 1.138 (0.541–2.392) | 0.733 | 1.127 (0.533–2.384) | 0.755 |
| Two or more causes | 0.955 (0.428–2.133) | 0.912 | 0.725 (0.320–1.642) | 0.441 |
| Non-stenotic (< 50%)  relevant artery plaque | 1.143 (0.434–3.008) | 0.787 | 0.843 (0.317–2.241) | 0.732 |
| Complex aortic plaque | 2.520 (1.075–5.907) | 0.033 | 1.741 (0.718–4.218) | 0.220 |

Cox proportional hazards regression analysis was conducted after adjusting for variables that were significant (p<0.05) in the univariable analysis along with the ESUS subtype.

HR, hazard ratio; CI, confidence interval; NIHSS, National Institutes of Health Stroke Scale; HDL, high-density lipoprotein cholesterol; LDL, low-density lipoprotein cholesterol; Ref, reference.

# Supplemental Table 3. Univariable and multivariable analysis for the factor associated with hemorrhagic stroke occurrence

|  | Univariable analysis | | Multivariable analysis | |  |  |
| --- | --- | --- | --- | --- | --- | --- |
|  | HR (95% CI) | P value | HR (95% CI) | P value |  |  |
| Demographics |  |  |  |  |  |  |
| Age, year | 1.011 (0.971–1.052) | 0.594 |  |  |  |  |
| Sex, men | 0.882 (0.314–2.479) | 0.812 |  |  |  |  |
| Risk factors |  |  |  |  |  |  |
| Hypertension | 1.175 (0.374–3.693) | 0.783 |  |  |  |  |
| Diabetes | 1.839 (0.654–5.166) | 0.248 |  |  |  |  |
| Dyslipidemia | 1.178 (0.332–4.183) | 0.800 |  |  |  |  |
| Current smoker | 0.200 (0.026–1.525) | 0.121 |  |  |  |  |
| Previous stroke | 4.186 (1.489–11.765) | 0.007 | 3.739 (1.310–10.675) | 0.014 |  |  |
| Initial NIHSS score | 1.074(0.968–1.192) | 0.180 |  |  |  |  |
| Laboratory findings |  |  |  |  |  |  |
| White blood cell | 1.000 (1.000–1.000) | 0.201 |  |  |  |  |
| Platelet | 0.994 (0.986–1.003) | 0.189 |  |  |  |  |
| Total cholesterol | 0.997 (0.986–1.009) | 0.616 |  |  |  |  |
| Triglyceride | 0.999 (0.992–1.006) | 0.764 |  |  |  |  |
| HDL | 0.998 (0.954–1.043) | 0.915 |  |  |  |  |
| LDL | 0.993 (0.979–1.008) | 0.378 |  |  |  |  |
| ESUS classification |  |  |  |  |  |  |
| Minor cardioembolic source | Ref. | 0.235 | Ref. | 0.272 |  |  |
| No cause | 3.011 (0.313–28.977) | 0.340 | 2.869 (0.298–27.608) | 0.362 |  |  |
| Two or more causes | 6.872 (0.827–57.100) | 0.074 | 5.554 (0.661–46.668) | 0.114 |  |  |
| Non-stenotic (< 50%)  relevant artery plaque | 4.970 (0.450–54.855) | 0.191 | 4.185 (0.377–46.484) | 0.244 |  |  |
| Complex aortic plaque | 10.523 (1.093–101.341) | 0.042 | 10.299 (1.070–99.157) | 0.044 |  |  |

Cox proportional hazards regression analysis was conducted after adjusting for variables that were significant (p<0.05) in the univariable analysis along with the ESUS subtype.

HR, hazard ratio; CI, confidence interval; NIHSS, National Institutes of Health Stroke Scale; HDL, high-density lipoprotein cholesterol; LDL, low-density lipoprotein cholesterol; Ref, reference.

# Supplemental Table 4. Univariable and multivariable analysis for the factor associated with mortality

|  | Univariable analysis | | Multivariable analysis | |
| --- | --- | --- | --- | --- |
|  | HR (95% CI) | P value | HR (95% CI) | P value |
| Demographics |  |  |  |  |
| Age, year | 1.057 (1.029–1.086) | < 0.001 | 1.043 (1.014–1.074) | 0.004 |
| Sex, men | 1.651 (0.855–3.188) | 0.136 |  |  |
| Risk factors |  |  |  |  |
| Hypertension | 2.415 (1.079–5.403) | 0.032 | 1.527 (0.649–3.596) | 0.332 |
| Diabetes | 2.111 (1.178–3.781) | 0.012 | 1.669 (0.909–3.064) | 0.098 |
| Dyslipidemia | 0.999 (0.466–2.144) | 0.998 |  |  |
| Current smoker | 0.503 (0.225–1.126) | 0.094 |  |  |
| Previous stroke | 1.710 (0.848–3.446) | 0.134 |  |  |
| Initial NIHSS score | 1.109 (1.055–1.165) | <0.001 | 1.083 (1.031–1.138) | 0.002 |
| Laboratory findings |  |  |  |  |
| White blood cell | 1.000 (1.000–1.000) | 0.441 |  |  |
| Platelet | 0.995 (0.991–1.000) | 0.068 |  |  |
| Total cholesterol | 0.998 (0.992–1.004) | 0.531 |  |  |
| Triglyceride | 0.998 (0.993–1.002) | 0.308 |  |  |
| HDL | 1.002 (0.980–1.025) | 0.840 |  |  |
| LDL | 0.997 (0.989–1.005) | 0.524 |  |  |
| ESUS classification |  |  |  |  |
| Minor cardioembolic  source | Ref | 0.079 | Ref | 0.455 |
| No cause | 1.624 (0.673–3.921) | 0.281 | 1.494 (0.604–3.696) | 0.385 |
| Two or more causes | 1.562 (0.628–3.885) | 0.337 | 1.176 (0.467–2.963) | 0.730 |
| Non-stenotic (< 50%)  relevant artery plaque | 1.555 (0.509–4.757) | 0.439 | 1.136 (0.368–3.500) | 0.825 |
| Complex aortic plaque | 3.852 (1.485–9.997) | 0.006 | 2.339 (0.869–6.292) | 0.092 |

Cox proportional hazards regression analysis was conducted after adjusting for variables that were significant (p<0.05) in the univariable analysis along with the ESUS subtype.

HR, hazard ratio; CI, confidence interval; NIHSS, National Institutes of Health Stroke Scale; HDL, high-density lipoprotein cholesterol; LDL, low-density lipoprotein cholesterol; Ref, reference.

**Supplemental Table 5.** **Predictive value of the ESUS subtype classification for classical risk factors**

|  | Classical risk factors alone | ESUS subtype  included | p-value |
| --- | --- | --- | --- |
|  | Predict ability (95% CI) | |  |
| Harrell’s c index | 0.665(0.618-0.713) | 0.684(0.64-0.727) | 0.1922 |
| Heagerty's iAUC | 0.662(0.618-0.706) | 0.679(0.638-0.719) | 0.1355 |
| Heagerty's Incident/ DynamicAUC (median follow up) | 0.697(0.636-0.758) | 0.72(0.664-0.775) | 0.1891 |
| Heagerty's Incident/ DynamicAUC (last follow up) | 0.736(0.657-0.815) | 0.746(0.672-0.82) | 0.5811 |
| NRI (median follow up) | - | 0.157(-0.015-0.27) | 0.068 |
| IDI (median follow up) | - | 0.012(0.003-0.048) | 0.016 |
| NRI (last follow up) | - | 0.534(-0.502-0.749) | 0.136 |
| IDI (last follow up) | - | 0.078(-0.003-0.176) | 0.068 |

AUC; Area Under the Curve, NRI; Net Reclassification Index, IDI; Integrated Discrimination Index.

**Supplemental Table 6. Etiologic evaluations according to ESUS subtypes**

|  | Total (n = 775) | Arteriogenic embolism (n= 161) | Minor cardioembolic source (n = 205) | No cause (n = 225) | Two or more causes (n = 184) | *p* value |
| --- | --- | --- | --- | --- | --- | --- |
| Angiographic evaluations | 746 (96.3%) | 154 (95.7%) | 202 (98.5%) | 207 (92.0%) | 183 (99.5%) | < 0.001 |
| CTA | 556 (71.7%) | 114 (70.8%) | 156 (76.1%) | 146 (64.9%) | 140 (76.1%) | 0.030 |
| MRA | 692 (89.3%) | 140 (87.0%) | 191 (93.2%) | 190 (84.4%) | 171 (92.9%) | 0.007 |
| DSA | 98 (12.6%) | 24 (14.9%) | 20 (9.8%) | 26 (11.6%) | 28 (15.2%) | 0.303 |
| Neurosonographic evaluations | 743 (95.9%) | 153 (95.0%) | 200 (97.6%) | 208 (92.4%) | 182 (98.9%) | 0.005 |
| Carotid Doppler | 686 (88.5%) | 143 (88.8%) | 177 (86.3%) | 196 (87.1%) | 170 (92.4%) | 0.248 |
| TCD | 699 (90.2%) | 140 (87.0%) | 193 (94.1%) | 197 (87.6%) | 169 (91.8%) | 0.049 |
| ABI | 775 (100) | 161 (100) | 205 (100) | 225 (100) | 184 (100) | NA |
| Echocardiography |  |  |  |  |  |  |
| TEE | 775 (100.0%) | 161 (100.0%) | 205 (100.0%) | 225 (100.0%) | 184 (100.0%) | NA |
| TTE | 694 (89.5%) | 145 (90.1%) | 194 (94.6%) | 187 (83.1%) | 168 (91.3%) | 0.001 |
| Prolonged heart rhythm evaluations | 775 (100.0%) | 161 (100.0%) | 205 (100.0%) | 225 (100.0%) | 184 (100.0%) | NA |
| Continuous ECG monitoring | 709 (91.5%) | 148 (91.9%) | 185 (90.2%) | 205 (91.1%) | 171 (92.9%) | 0.806 |
| Holter | 419 (54.1%) | 85 (52.8%) | 113 (55.1%) | 139 (61.8%) | 82 (44.6%) | 0.007 |
| Implantable loop recorder | 9 (1.2%) | 2 (1.2%) | 1 (0.5%) | 6 (2.7%) | 0 (0.0%) | 0.060 |
| Heart CT | 499 (64.4%) | 91 (56.5%) | 157 (76.6%) | 133 (59.1%) | 118 (64.1%) | < 0.001 |

ABI = ankle-brachial index; CT = computed tomography; CTA = computed tomography angiography; DSA = digital subtraction angiography; ECG = electrocardiogram; NA = not analyzed; TCD = transcranial Doppler; TEE = transesophageal echocardiography; TTE = transthoracic echocardiography. Data are expressed as number (%).
